# Facial Neuropathy Revealed: A Case Report on Trigeminal Schwannoma

**DOI:** 10.7759/cureus.52302

**Published:** 2024-01-15

**Authors:** Emeka Emedike, Azzam Alnughaythir, Naif Alsayed, Ahlam Alharbi, Bader Alotibi

**Affiliations:** 1 Radiology, Dallah Hospital, Riyadh, SAU; 2 General Practice, Shaqra University, Shaqra, SAU; 3 Family Medicine, Primary Health Care Center, Riyadh, SAU

**Keywords:** magnetic resonance imaging, neuropathy, schwannoma, trigeminal nerve, facial numbness

## Abstract

Trigeminal schwannomas, rare tumors originating from Schwann cells of the trigeminal nerve, present unique diagnostic challenges due to their infrequent occurrence. The clinical manifestation of facial numbness and tingling necessitates a comprehensive approach for accurate diagnosis and optimal management. We present the case of a 45-year-old female who presented with a six-month history of progressive facial numbness localized to the left maxillary and mandibular regions. Intermittent sharp, shooting pain exacerbated by chewing and cold stimuli was reported. Physical examination revealed sensory deficits in left trigeminal nerve distribution. Magnetic resonance imaging confirmed a well-circumscribed, enhancing lesion along the left trigeminal nerve. Surgical excision of the tumor confirmed the diagnosis of schwannoma. This case underscores the significance of a detailed clinical history, advanced imaging, and collaboration between neurologists and neurosurgeons in achieving an accurate diagnosis and favorable outcome for trigeminal schwannomas. The successful surgical intervention, coupled with histopathological confirmation, contributes to the understanding of these rare tumors.

## Introduction

Trigeminal schwannomas represent an exceedingly rare subset of intracranial neoplasms originating from Schwann cells of the trigeminal nerve [1]. They constitute one-third of Meckel's cave tumors while representing less than 0.2% of all intracranial tumors [1,2]. These lesions present unique diagnostic challenges due to their infrequency and subtle clinical onset. The trigeminal nerve, responsible for facial sensation, can be affected by various pathologies, making the differentiation between these entities crucial for accurate diagnosis and appropriate management [1,2].

The clinical presentation of trigeminal schwannomas often involves sensory disturbances, such as facial numbness and tingling, which can be mistaken for other trigeminal nerve disorders. The insidious growth of these tumors may lead to a delayed diagnosis, necessitating a comprehensive approach involving detailed clinical history, meticulous physical examination, and advanced imaging modalities [2]. This case report contributes to the existing body of literature by providing a detailed exploration of a patient with schwannoma of the right trigeminal nerve, emphasizing the complexities involved in diagnosis, differential considerations, and the multidisciplinary approach essential for optimal patient outcomes.

## Case presentation

A 45-year-old female presented to the neurology outpatient clinic reporting a six-month history of progressive facial numbness localized to the left maxillary and mandibular regions. The sensation was described as intermittent tingling, accompanied by occasional sharp, shooting pain exacerbated by chewing and exposure to cold stimuli. The onset was insidious, without identifiable triggers, and the symptoms gradually intensified over time.

Upon thorough inquiry, the patient denied any history of trauma, recent infections, or exposure to environmental toxins. Medical and family histories were unremarkable, with no reported neurologic conditions. The patient did not present with associated visual disturbances, motor weakness, or alterations in taste perception.

Physical examination revealed diminished sensation to light touch and pinprick over the left maxillary and mandibular divisions of the trigeminal nerve. The corneal reflex was intact, and no signs of facial weakness, atrophy, or asymmetry were observed. The remainder of the cranial nerve examination, as well as the general physical examination, yielded unremarkable findings.

Laboratory investigations, including complete blood count, basic metabolic panel, and inflammatory markers, were within normal limits (Table 1). The cerebrospinal fluid analysis did not reveal any abnormalities. The differential diagnosis considered trigeminal neuralgia, multiple sclerosis, and other space-occupying lesions affecting the left trigeminal nerve.

**Table 1:** Laboratory test results with reference range CSF: cerebrospinal fluid

Lab Test	Result (Patient)	Reference Range
White Blood Cell Count	7.2 x 10^3^/μL	4.0–11.0 x 10^3^/μL
Hemoglobin	13.8 g/dL	12.0–16.0 g/dL
Platelet Count	250 x 10^3^/μL	150–400 x 10^3^/μL
Sodium	138 mmol/L	135–145 mmol/L
Potassium	4.2 mmol/L	3.5–5.0 mmol/L
Blood Urea Nitrogen	12 mg/dL	7–20 mg/dL
Creatinine	0.8 mg/dL	0.6–1.1 mg/dL
C-Reactive Protein	0.5 mg/L	0.0–3.0 mg/L
CSF Cell Count	3 cells/μL	0–5 cells/μL
CSF Protein	35 mg/dL	15–45 mg/dL
CSF Glucose	60 mg/dL	40–70 mg/dL

**Figure 1:**
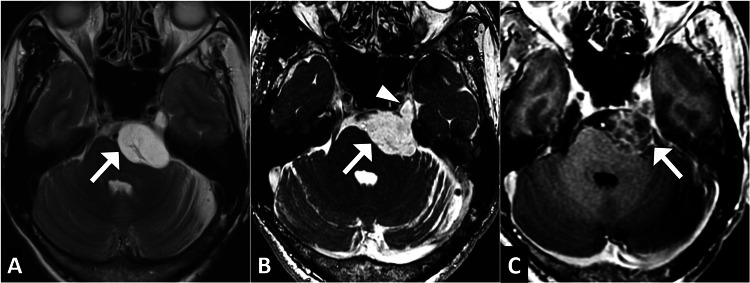
Axial brain MRI images in T2-weighted image (A), CISS image (B), and T1-post contrast image (C) showing a well-defined lesion (arrow) at the left cerebellopontine angle extending into the left Meckel's cave (arrowhead), suggestive of trigeminal schwannoma CISS: constructive interference in steady state image, MRI: magnetic resonance imaging

In light of the clinical presentation, advanced imaging was pursued. Magnetic resonance imaging of the brain with gadolinium contrast revealed a well-circumscribed, enhancing lesion along the left trigeminal nerve, consistent with a schwannoma. The lesion measured approximately 2.5 cm in diameter and exhibited a hypo-intense signal on T1-weighted images and a hyper-intense signal on T2-weighted images (Figure 1).

**Figure 2:**
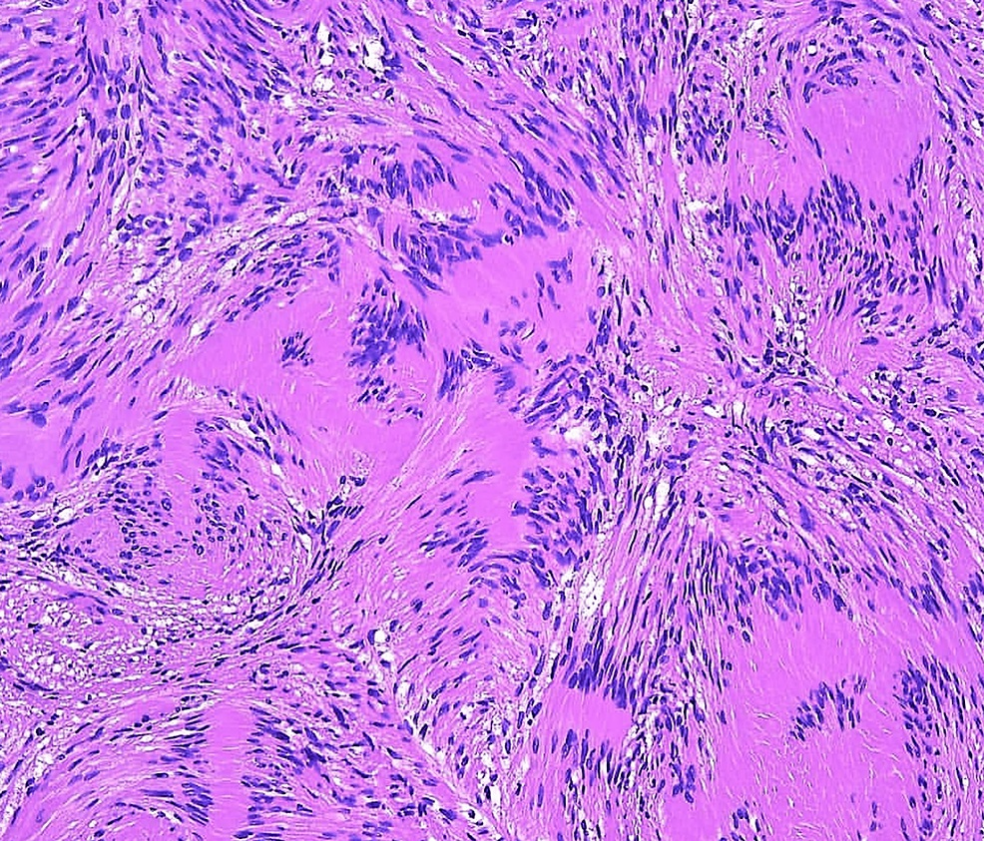
Histopathological examination of the excised tissue revealing spindle cells characteristic of schwannoma

The final diagnosis of schwannoma of the left trigeminal nerve was confirmed through comprehensive clinical correlation and exclusion of alternative etiologies. Given the size and location of the lesion, a multidisciplinary team comprising neurosurgeons and neurologists was involved in the management. A surgical resection of the schwannoma was performed, and a histopathological examination postoperatively confirmed the diagnosis (Figure 2).

The patient's postoperative course was uneventful, with a gradual resolution of facial numbness and significant improvement in symptoms. Follow-up imaging confirmed complete excision of the lesion, affirming the success of the intervention and the overall positive trajectory of the patient's recovery.

## Discussion

The presented case of a schwannoma involving the right trigeminal nerve underscores the intricacies associated with the diagnosis and management of rare neurologic tumors. Trigeminal schwannomas are exceptionally rare, comprising a minute proportion of intracranial neoplasms, and their manifestation along the trigeminal nerve poses unique challenges. The clinical presentation, characterized by progressive facial numbness and intermittent pain, reflects the insidious growth of these tumors and highlights the importance of a meticulous clinical evaluation [3,4].

The accurate diagnosis of trigeminal schwannomas necessitates a comprehensive approach, including a detailed clinical history, thorough neurological examination, and advanced imaging studies. In this case, magnetic resonance imaging with gadolinium contrast played a pivotal role in delineating the anatomical extent of the lesion and guiding the subsequent management strategy [2-5].

Differential diagnosis in cases of trigeminal nerve involvement must consider various pathologies, including trigeminal neuralgia, multiple sclerosis, and other space-occupying lesions. The exclusion of these alternatives through a combination of clinical correlation and laboratory investigations further solidified the diagnosis in our case. Notably, the absence of motor deficits and the intact corneal reflex were consistent with the classical presentation of trigeminal schwannomas, aiding in the differentiation from other potential etiologies [1,3].

The multidisciplinary approach to management, involving collaboration between neurologists and neurosurgeons, ensured a comprehensive evaluation and optimal treatment strategy. Surgical resection remains the mainstay of treatment for trigeminal schwannomas, aiming for complete excision while preserving neurological function [2-4]. In this case, the successful surgical intervention led to the resolution of facial numbness and improvement in the patient's overall quality of life.

Histopathological examination post-surgery confirmed the diagnosis of schwannoma, further supporting the preoperative clinical and radiological findings. The benign nature of schwannomas, as evidenced by the absence of atypical cellular features, aligns with existing literature on these tumors and contributes to the overall favorable prognosis associated with their management [2,5].

## Conclusions

The presented case of a left trigeminal schwannoma exemplifies the complexities inherent in diagnosing and managing rare neurologic tumors. The successful multidisciplinary approach, incorporating detailed clinical evaluation, advanced imaging, and surgical intervention, underscores the importance of a comprehensive strategy in addressing such cases. The positive outcome following surgical resection, confirmed by histopathological examination, not only validates the diagnosis but also emphasizes the effectiveness of timely and targeted interventions. This case contributes to the growing body of knowledge on trigeminal schwannomas, highlighting the need for ongoing research to enhance diagnostic precision and therapeutic modalities. Ultimately, the collaborative efforts of neurologists, neurosurgeons, and pathologists in navigating the intricacies of this case serve as a testament to the significance of interdisciplinary approaches in ensuring optimal outcomes for patients with rare neurologic conditions.
